# Thyroid Hormones and Type 2 Diabetes Mellitus: A Narrative Review of Findings From 18 Years of Follow-up in the Tehran Thyroid Study

**DOI:** 10.5812/ijem-167206

**Published:** 2026-04-29

**Authors:** Ladan Mehran, Mohamadamin Tarighat-Payma, Safdar Masoumi, Alireza Amirabadizadeh, Fereidoun Azizi, Atieh Amouzegar

**Affiliations:** 1 Endocrine Research Center, Research Institute for Endocrine Disorders, Research Institute for Endocrine Sciences, Shahid Beheshti University of Medical Sciences, Tehran, Iran; 2 Medical Toxicology and Drug Abuse Research Center (MTDRC), Birjand University of Medical Sciences, Birjand, Iran

**Keywords:** Tehran Thyroid Study, Type 2 Diabetes Mellitus, Fasting Plasma Glucose, Thyroid Function, Subclinical Thyrotoxicosis, Thyroid Dysfunction, Hypothyroidism, Hyperthyroidism, Thyroid Hormones

## Abstract

**Context:**

Thyroid dysfunction and type 2 diabetes mellitus (T2DM) are common endocrine disorders with substantial public health implications. Increasing evidence suggests that alterations in thyroid function may influence glycemic regulation and the risk of T2DM. However, long-term population-based evidence on this relationship remains limited. Therefore, this study aimed to summarize the association between thyroid function and T2DM using findings from the Tehran Thyroid Study (TTS), a large community-based cohort with nearly 2 decades of follow-up.

**Evidence Acquisition:**

A literature search was performed in PubMed, Scopus, Web of Science, and the library of the Research Institute for Endocrine Sciences to identify articles within the TTS framework. The retrieved articles were categorized according to their primary predictors and outcomes, including T2DM, thyroid function, thyroid hormone sensitivity, and insulin resistance.

**Results:**

Over 18 years, a longitudinal analysis of 1938 adults showed that each unit increase in log-transformed thyroid-stimulating hormone (TSH) was associated with a 25% lower risk of developing T2DM (HR, 0.75; 95% CI, 0.64 - 0.90), whereas higher free thyroxine (FT4) levels were associated with a slightly increased risk of T2DM. Cross-sectional data indicated that overt and subclinical hyperthyroidism significantly increased the odds of hyperglycemia, with the highest prevalence (31.3%) observed in subclinical hyperthyroidism. In studies of thyroid hormone sensitivity, a 1-SD increase in the thyroid feedback quantile-based index (TFQI) was associated with higher odds of T2DM, whereas higher central sensitivity indices were associated with lower odds of prediabetes. Lower FT4 levels were independently associated with higher insulin resistance in euthyroid men but not in women. Patients with diabetes had a higher prevalence of subclinical hyperthyroidism but a lower incidence of thyroid dysfunction compared with controls.

**Conclusions:**

Trends toward lower TSH or relatively higher FT4 levels are associated with an increased incidence of T2DM. Both overt and subclinical hyperthyroidism are associated with hyperglycemia. Current evidence supports targeted metabolic monitoring in individuals with thyroid hormone excess or altered thyroid sensitivity indices but does not justify routine thyroid-directed therapy solely to prevent T2DM.

## 1. Context

Thyroid disorders and type 2 diabetes mellitus (T2DM) are among the most prevalent endocrine diseases worldwide and account for a substantial proportion of morbidity, mortality, and health care costs. The global burden of T2DM is increasing, with current estimates indicating that more than 500 million people have T2DM, particularly in low- and middle-income countries (1). Thyroid dysfunction, including overt and subclinical forms, is also common and is associated with a wide range of metabolic, cardiovascular, and neuropsychiatric outcomes (2, 3). Given the high prevalence of these conditions and the overlap in their pathophysiological mechanisms, the coexistence of thyroid dysfunction and T2DM has long been a focus of clinical and research interest (4, 5, 6).

Accumulating evidence indicates that thyroid status may influence glucose homeostasis, insulin resistance, and T2DM risk. Conversely, T2DM and impaired glucose metabolism may predispose individuals to thyroid dysfunction (6, 7, 8). However, the direction and strength of these relationships remain controversial. Although some studies suggest that subtle variations in thyroid function within the euthyroid range are associated with glycemic outcomes, others do not support such associations (9, 10). Moreover, the clinical relevance of these findings is complicated by heterogeneity across populations, sex-specific differences, and challenges in interpreting novel thyroid hormone sensitivity indices. Notably, it remains unclear whether the detection and treatment of subclinical thyroid dysfunction can prevent T2DM onset or improve glycemic control (11).

The Tehran Thyroid Study (TTS) is a large population-based cohort with more than 18 years of follow-up, providing a unique opportunity to clarify these bidirectional associations (12). The TTS has generated a series of original investigations using diverse analytic approaches, including cross-sectional analyses, longitudinal models, and advanced joint longitudinal/time-to-event methods, based on repeated measurements of thyroid function tests, thyroid autoantibodies, thyroid hormone sensitivity indices, and metabolic outcomes (13). This review aimed to summarize the existing findings from 18 years of TTS follow-up regarding the bidirectional relationship between thyroid function and T2DM and to discuss clinical implications, limitations, and directions for future research.

## 2. Evidence Acquisition

**Figure 1.**
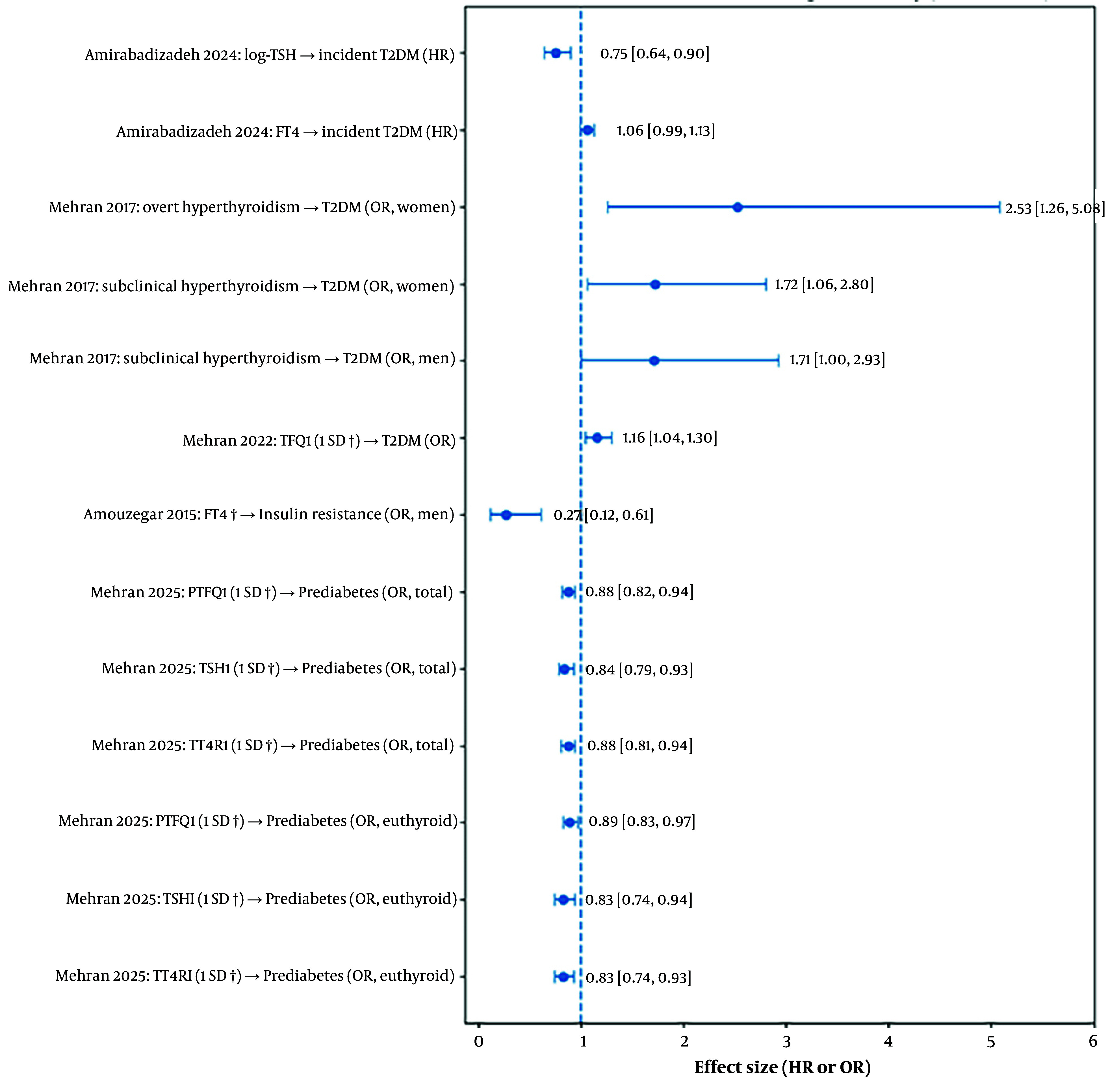
Forest plot of effect sizes for thyroid function and the risk of type 2 diabetes mellitus (T2DM), prediabetes, or insulin resistance. The plot presents hazard ratios (HRs) or odds ratios (ORs) with 95% confidence intervals (CIs) for each association.

For this narrative review, we focused exclusively on studies conducted within the TTS framework. Relevant publications were identified through a targeted search of PubMed, Scopus, Web of Science, and the library of the Research Institute for Endocrine Sciences. We included original TTS articles that examined any aspect of the association between thyroid function, including TSH, FT4, thyroid autoantibodies, and thyroid hormone sensitivity indices, and glycemic outcomes, including fasting plasma glucose (FPG), insulin resistance, prediabetes, and T2DM. Based on these criteria, 8 publications were identified (14, 15, 16, 17, 18, 19, 20, 21). These included cross-sectional analyses (n = 5; 2 studies used thyroid sensitivity indices) and cohort analyses (n = 3; 1 study used a joint longitudinal/time-to-event cohort analysis). A summary of these studies and their findings is presented in Table 1 and Figure 1.

**Table 1.** Studies Conducted in the Tehran Thyroid Study on the Association Between Thyroid Function and Type 2 Diabetes Mellitus (14, 15, 18-20)

Study	Year	Design	Population	Follow-up Years	Exposures/Metrics	Main Findings
**Amirabadizadeh et al. (14)**	2024	Cohort; joint longitudinal and time-to-event analysis	1938	> 10	Longitudinal TSH and FT4	An increase in log-TSH over time was associated with lower T2DM incidence (HR, 0.75; 95% CI, 0.64 - 0.90); increased FT4 was marginally associated with higher risk (HR, 1.06).
**Mehran et al. (15)**	2017	Cross-sectional; population-based	5422	—	TSH, FT4, and clinical thyroid states	Overt and subclinical hyperthyroidism were associated with higher odds of hyperglycemia; the highest prevalence was observed in subclinical hyperthyroidism, with a prevalence of 31.3%.
**Mehran et al. (16)**	2014	Cross-sectional; euthyroid population	3755	—	FT4 and TSH	No linear association with FPG was observed; high FPG decreased across FT4 tertiles, from 13.3% in the lowest tertile to 10.0% in the highest tertile.
**Mehran et al. (17)**	2017	Longitudinal; generalized estimating equations	2793	10	TSH and FT4 within the reference range	Variations in TSH and FT4 were not significantly associated with the high-FPG component of metabolic syndrome.
**Mehran et al. (18)**	2022	Cross-sectional	5124	—	TFQI, PTFQI, TT4RI, and TSHI	A 1-SD increase in TFQI was associated with higher odds of T2DM in euthyroid participants (OR, 1.16); TSHI and TT4RI were not associated with T2DM.
**Mehran et al. (19)**	2025	Cross-sectional	4356	—	PTFQI, TSHI, and lnTT4RI	A 1-SD increase in PTFQI, TSHI, and lnTT4RI was associated with lower odds of prediabetes; PTFQI, TSHI, and lnTT4RI had negative correlations with FPG and 2-hour plasma glucose.
**Amouzegar et al. (20)**	2015	Cross-sectional; euthyroid population	2758	—	TSH, FT4, TPOAb, and HOMA-IR	In men, lower FT4 was associated with higher HOMA-IR (β = -0.09) and higher odds of insulin resistance (OR, 0.27); no significant association was observed in women.
**Gholampour Dehaki et al. (21)**	2017	Cohort; individuals with diabetes vs prediabetes vs controls	435 / 286 / 989	11	TSH, TPOAb, and incident thyroid disease	Thyroid dysfunction was significantly more frequent in individuals with prediabetes and diabetes, but this association was no longer significant after adjustment. Subclinical hyperthyroidism was more frequent in T2DM (OR, 1.84), and the incidence of subclinical hypothyroidism was lowest in T2DM (OR, 0.47).

## 3. Results

### 3.1. Effect of Thyroid Hormones on Type 2 Diabetes Mellitus and Fasting Plasma Glucose

The effect of thyroid hormones on T2DM and dysglycemia has been investigated in the TTS using various methodologies (14, 15, 16, 17). A cohort study of 1938 individuals with more than a decade of follow-up used a novel joint model of longitudinal and time-to-event data to assess the effect of changes in thyroid hormone levels on the incidence of T2DM. This analysis showed that each unit increase in log-transformed TSH over time was associated with a 25% decrease in T2DM incidence (HR, 0.75; 95% CI, 0.64 - 0.90; P = 0.003), whereas each unit increase in FT4 was marginally associated with a higher risk (HR, 1.06; 95% CI, 0.99 - 1.13; P = 0.06). Therefore, a longitudinal trend toward hyperthyroidism, particularly a downward trend in TSH values, was associated with the development of T2DM (14).

The findings of this study are consistent with those of another cross-sectional study of 5422 individuals, which reported that overt and subclinical hyperthyroidism were associated with significantly higher odds of hyperglycemia. In women, the odds ratio for hyperglycemia in the presence of overt hyperthyroidism was 2.53 (95% CI, 1.26 - 5.08), and the odds ratio for subclinical hyperthyroidism was 1.72 (95% CI, 1.06 - 2.80). Similarly, in men with subclinical hyperthyroidism, the odds ratio for hyperglycemia was 1.71 (95% CI, 1.00 - 2.93). However, the association with overt hyperthyroidism was not statistically significant because of the small sample size. The highest prevalence was observed in subclinical hyperthyroidism, with a prevalence of 31.3% (P < 0.001) (15).

In another cross-sectional study of 3755 euthyroid participants, FPG was not associated with TSH or FT4 in linear regression analysis. However, the prevalence of high FPG decreased significantly from 13.3% in the lowest FT4 tertile to 10.0% in the highest FT4 tertile (16). In contrast, a 10-year longitudinal follow-up study of 2793 individuals that used generalized estimating equations found that variations in TSH or FT4 over time were not significantly associated with the high-FPG component of metabolic syndrome (17).

### 3.2. Thyroid Hormone Sensitivity and Type 2 Diabetes Mellitus

Two cross-sectional studies from the TTS investigated the association between thyroid hormone sensitivity and T2DM (18, 19). The first study included 5124 participants and calculated thyroid hormone resistance using 4 indices: TFQI, PTFQI, TT4RI, and TSHI. Among euthyroid participants, a 1-SD increase in TFQI was significantly associated with T2DM (OR, 1.16; 95% CI, 1.04 - 1.30; P = 0.009). This association was also present for known T2DM (OR, 1.18; 95% CI, 1.03 - 1.35; P = 0.001) but not for new-onset T2DM. TSHI and TT4RI were not associated with T2DM in euthyroid participants (18).

The second study included 4356 participants and evaluated the association between thyroid hormone sensitivity and prediabetes using PTFQI, TSHI, and lnTT4RI. A 1-SD increase in all 3 indices was significantly associated with lower odds of prediabetes in the total population and the euthyroid subgroup. In the total population, the ORs were 0.88 (95% CI, 0.82 - 0.94) for a 1-SD increase in PTFQI, 0.84 (95% CI, 0.79 - 0.93) for a 1-SD increase in TSHI, and 0.88 (95% CI, 0.81 - 0.94) for a 1-SD increase in TT4RI. In the euthyroid population, the corresponding ORs were 0.89 (95% CI, 0.83 - 0.97), 0.83 (95% CI, 0.74 - 0.94), and 0.83 (95% CI, 0.74 - 0.93), respectively. This study also found negative correlations between all 3 sensitivity indices and both FPG and 2-hour plasma glucose levels (PTFQI, r = -0.096; TSHI, r = -0.054; lnTT4RI, r = -0.031) (19).

### 3.3. Thyroid Hormones and Insulin Resistance

A cross-sectional study from the TTS investigated the association between thyroid function and homeostatic model assessment for insulin resistance (HOMA-IR) in 2758 euthyroid individuals (20). In men, a significant negative association was observed between FT4 and HOMA-IR (β = -0.09; P < 0.001), indicating that lower FT4 levels, even within the normal range, were independently associated with a higher likelihood of insulin resistance (OR, 0.27; 95% CI, 0.12 - 0.61; P < 0.01). Serum TSH showed a positive association with HOMA-IR in linear regression analysis in men (β = 0.07; P = 0.006). This association was no longer significant in logistic regression. In contrast, no significant association was observed between FT4, TSH, and HOMA-IR in women. Furthermore, the study found no significant differences in HOMA-IR or FPG between individuals who were positive or negative for thyroid peroxidase antibodies (TPOAb).

### 3.4. Effect of Type 2 Diabetes Mellitus on Thyroid Function

A cohort study from the TTS compared the prevalence and incidence of thyroid disorders among 435 individuals with T2DM, 286 individuals with prediabetes, and 989 healthy controls over 11 years of follow-up (21). The crude prevalence of thyroid dysfunction was 18.9% in individuals with diabetes, 19.3% in individuals with prediabetes, and 13.5% in controls (unadjusted P = 0.01). However, no significant difference was found among the 3 groups after adjustment for age, sex, smoking, blood pressure, body mass index (BMI), TPOAb, TSH, insulin resistance index, triglycerides, and cholesterol. In the prevalence analysis, subclinical hyperthyroidism was more frequent in individuals with diabetes than in controls (OR, 1.84; 95% CI, 1.06 - 3.18; P = 0.02). Overt hypothyroidism was less frequent in individuals with prediabetes than in controls (OR, 0.42; 95% CI, 0.18 - 0.98; P = 0.04). The incidence rates of thyroid dysfunction were 14, 18, and 21 per 1000 person-years in individuals with diabetes, individuals with prediabetes, and controls, respectively. The incidence of thyroid dysfunction was lower in individuals with diabetes than in controls (OR, 0.59; 95% CI, 0.36 - 0.95; P = 0.03). However, this association was no longer significant after adjustment for covariates. The incidence of subclinical hypothyroidism remained significantly lower in individuals with diabetes than in controls (OR, 0.47; 95% CI, 0.25 - 0.89; P = 0.02).

## 4. Conclusions

The TTS has demonstrated a bidirectional and complex association between thyroid physiology and T2DM over 18 years. Longitudinal trends toward lower TSH and relatively higher FT4 levels were associated with an increased incidence of T2DM in joint longitudinal/time-to-event analyses. In addition, cross-sectional and cohort analyses from the TTS demonstrated that both overt and subclinical hyperthyroid states are associated with a higher prevalence of hyperglycemia and an increased incidence of T2DM (14, 15). Lower FT4 values were associated with insulin resistance and components of metabolic syndrome in sex-specific analyses within the euthyroid range (16, 17, 20). Novel indices of central and peripheral thyroid hormone sensitivity, including TFQI, PTFQI, TSHI, and TT4RI, derived within the TTS identified physiological variation associated with prevalent T2DM and prediabetes, suggesting that these metrics may capture clinically relevant endocrinologic heterogeneity not evident from TSH and FT4 alone (18, 19). Conversely, individuals with T2DM or prediabetes did not uniformly exhibit an increased adjusted incidence of thyroid dysfunction in the TTS, although specific patterns, such as a greater prevalence of subclinical hyperthyroidism among individuals with diabetes, warrant further investigation (21). Taken together, the TTS findings provide a consistent picture of heterogeneity, showing that dynamic longitudinal changes and static indices of thyroid function and sensitivity influence glycemic outcomes.

In the TTS, thyroid hormone sensitivity indices, including TFQI, PTFQI, TSHI, and TT4RI, have shown consistent associations with diabetic phenotypes in cross-sectional analyses. These findings indicate that variation in central feedback sensitivity or peripheral hormone responsiveness may have biological and clinical relevance (18, 19). Such indices may improve risk stratification among euthyroid individuals with TSH and FT4 values within the reference range. They may also serve as intermediate phenotypes for mechanistic investigation. However, most available data are observational and cross-sectional. Prospective studies are needed to validate these associations and determine the predictive value of these indices for incident T2DM.

Sex-stratified analyses in the TTS indicated that some associations, such as those between FT4 and HOMA-IR or insulin resistance, were stronger or present only in men (20). This finding is consistent with previous literature showing sex-specific variation in thyroid epidemiology and metabolic interactions (16, 20).

The apparent discrepancy between Amirabadizadeh et al. (14), who found an association between thyroid hormone changes and T2DM, and Mehran et al. (17), who found no association between thyroid hormone variations and high FPG, likely reflects differences in study methodology and design. The joint longitudinal/time-to-event model examines within-person trends and individual risk, whereas the generalized estimating equation approach assesses population-average associations. Differences in outcomes, analytic methods, sample size, and hormone variability likely explain these contradictory results.

The TTS findings align with several international studies that have also reported links between thyroid function and T2DM. In the Rotterdam Study, Chaker et al. observed that higher TSH, even within the reference range, was associated with a greater incidence of T2DM, whereas higher FT4 was protective (22). A meta-analysis of 12 prospective cohorts also indicated that elevated TSH was associated with a 17% higher risk of diabetes and that lower FT3 and FT4 were significantly associated with increased risk, with nonlinear, J-shaped, or inverted J-shaped relationships (9). Another systematic review found that hypothyroidism, whether clinical or subclinical, conferred a higher diabetes risk and that even lower-normal FT4 values were linked to T2DM incidence (8). Importantly, Mendelian randomization analyses have provided additional insights. Su et al. reported a causal association between genetically elevated TSH and hypothyroidism and T2DM, combining National Health and Nutrition Examination Survey data with genetic factors (23). Genetic analyses also show that higher normal-range FT4 may protect against metabolic syndrome, including raised FPG, and that TSH is associated with lipid abnormalities (24).

The physiological mechanisms linking thyroid status and glucose homeostasis are well established and multifactorial. Thyroid hormones increase hepatic glucose output, stimulate lipolysis and free fatty acid flux, alter insulin clearance and peripheral insulin sensitivity, and change body composition and energy expenditure. These pathways can accelerate dysglycemia in states of thyroid excess (7). In contrast, chronic insulin resistance, hyperinsulinemia, and metabolic and inflammatory changes associated with T2DM may affect hypothalamic-pituitary-thyroid axis set points and peripheral thyroid hormone metabolism. These mechanisms may help explain why the association between T2DM and new-onset thyroid disease is attenuated after statistical adjustment in the TTS cohort (7, 21).

### 4.1. Clinical Implications

Current TTS data support heightened clinical vigilance for dysglycemia in patients with overt or subclinical hyperthyroidism and suggest that thyroid variation may identify individuals at greater metabolic risk even within the reference range (14, 15, 16). Nevertheless, no available observational evidence, including evidence from the TTS, supports generalized thyroid treatment to prevent T2DM. Randomized clinical trials are required to determine whether treating subclinical thyroid dysfunction or modulating thyroid hormone sensitivity indices affects T2DM risk or glycemic control. In this regard, limited intervention studies of levothyroxine and insulin resistance are promising but remain inconclusive (11).

### 4.2. Limitations

Although the TTS has several strengths, including extended follow-up and repeated measurements, it also has limitations. These include confounding typical of observational designs, potential selection and survivor biases over extended follow-up, and limited power for rare exposures, such as overt hyperthyroidism, in some subgroup analyses (14, 15, 16, 17, 21). Furthermore, most studies of sensitivity indices are cross-sectional, and these indices have not been examined using repeated measurements in a cohort study. These indices require external validation in other ethnic and geographic populations before they can be widely used in clinical practice (18, 19).

### 4.3. Directions for Future Research

Future research should focus on several interconnected areas. First, prospective studies are needed to determine whether thyroid sensitivity indices can predict the development of T2DM beyond established risk scores. Second, mechanistic studies in humans and animal models are essential to clarify the causal pathways linking thyroid function dynamics with insulin action and beta-cell function. Third, randomized controlled trials should evaluate whether interventions targeting subclinical thyroid dysfunction or modulating thyroid hormone signaling can influence the incidence of T2DM. Finally, Mendelian randomization analyses using large genome-wide association study summary datasets and pooled genetic data from TTS participants and external datasets are needed to investigate the causal effects of TSH, FT4, and thyroid sensitivity indices on the risk of developing T2DM.

### 4.4. Key Messages

Over 18 years, the TTS showed that trends toward lower TSH and relatively higher FT4 levels are associated with an increased incidence of T2DM. Overt and subclinical hyperthyroidism are associated with hyperglycemia. Thyroid hormone sensitivity indices, including TFQI, PTFQI, TSHI, and TT4RI, capture physiological variation linked to T2DM and prediabetes that is not detected by conventional TSH or FT4 measurements. Current evidence supports targeted metabolic monitoring in patients with thyroid excess or abnormal sensitivity indices, but not routine thyroid treatment solely for the prevention of T2DM.

## Data Availability

This review is based on previously published data, which are available from the cited sources. Any datasets compiled or analyzed by the authors during the review are available from the corresponding author upon reasonable request.
